# Recent Trends in the Management of Acne Vulgaris: A Review Focusing on Clinical Studies in the Last Decade

**DOI:** 10.7759/cureus.56596

**Published:** 2024-03-20

**Authors:** Sharwari Jaiswal, Sugat Jawade, Bhushan Madke, Shreya Gupta

**Affiliations:** 1 Dermatology, Jawaharlal Nehru Medical College, Datta Meghe Institute of Higher Education and Research, Wardha, IND; 2 Dermatology, Datta Meghe Medical College, Datta Meghe Institute of Higher Education & Research, Wanadongri, IND

**Keywords:** patient-centered care, pathogenesis, treatment modalities, management, clinical studies, acne vulgaris

## Abstract

Acne vulgaris is a prevalent chronic inflammatory skin condition with significant implications for quality of life, particularly among adolescents and young adults. Recent advancements in understanding its pathophysiology and developing novel therapeutic modalities have reshaped the landscape of acne management. This review provides an overview of recent trends in acne management, focusing on clinical studies conducted in the past decade. Key findings include insights into acne pathogenesis, emerging treatment modalities, comparative effectiveness of traditional and emerging therapies, and considerations for patient-centered care. The review underscores the importance of staying updated with recent clinical studies to provide evidence-based care and optimize patient treatment outcomes. Moreover, it highlights the need for continued research efforts to develop personalized treatment approaches, explore combination therapies, and address the psychosocial impact of acne. Collaborative endeavors between clinicians and researchers are essential to advance the field of acne management and improve patient outcomes.

## Introduction and background

Acne vulgaris is a common chronic inflammatory skin condition affecting the pilosebaceous units [1]. It is characterized by forming comedones, papules, pustules, nodules, and cysts, primarily on the face, neck, chest, and back. Acne typically begins during adolescence due to increased sebum production, follicular hyperkeratinisation, bacterial colonization, and inflammation. Despite being prevalent among teenagers, acne can persist into adulthood and significantly impact the quality of life [2].

The management of acne vulgaris has evolved over the years with advancements in understanding its pathophysiology and developing novel therapeutic modalities. Keeping abreast of recent clinical studies is crucial for healthcare providers to provide evidence-based care and optimize patient treatment outcomes [3]. By reviewing recent research, clinicians can gain insights into emerging trends, efficacy, safety profiles, and comparative effectiveness of various treatment options [4].

This review aims to provide a comprehensive overview of recent trends in managing acne vulgaris, explicitly focusing on clinical studies conducted in the last decade. By synthesizing the latest evidence, this review intends to elucidate advancements in treatment modalities, identify emerging therapies, discuss their clinical efficacy and safety profiles, and highlight future directions in acne management. The ultimate goal is to equip healthcare professionals with up-to-date knowledge to deliver personalized, effective, patient-centered care for individuals with acne vulgaris.

## Review

Pathogenesis of acne vulgaris

Overview of Acne Pathophysiology

**Figure 1:**
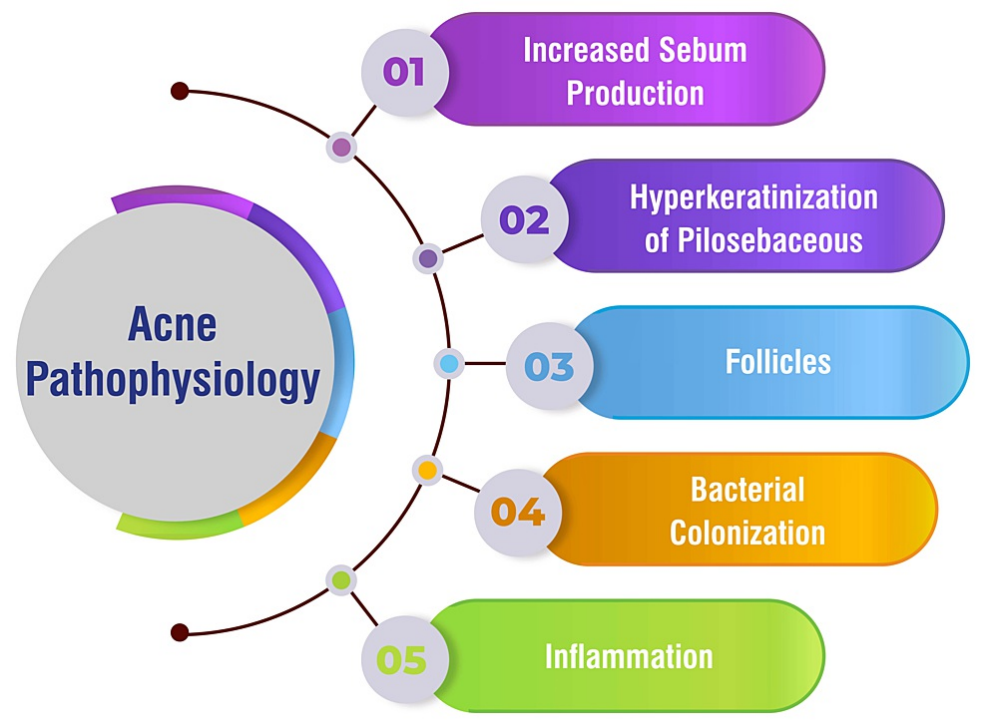
Acne pathophysiology. This figure is self-created by the corresponding author.

Acne vulgaris arises from a multifactorial interplay of physiological processes within the pilosebaceous units. First, there's a notable increase in sebum production within hair follicles, a prominent contributor to acne formation [1]. Additionally, hyperkeratinization of pilosebaceous follicles occurs, disrupting the normal shedding of skin cells and forming blackheads, further exacerbating acne development [1]. Moreover, the colonization of follicles by *Cutibacterium acnes*, formerly known as *Propionibacterium acnes*, is pivotal in acne pathogenesis, as it induces inflammation within the follicles, contributing to the progression of the condition [1]. Inflammation is central to acne development, manifesting as papules, pustules, and nodules characteristic of the condition [1]. Beyond these primary factors, the complex pathogenesis of acne vulgaris is influenced by genetics, environmental factors, hormonal imbalances, stress, and other variables [1]. Understanding these underlying mechanisms is paramount for successfully developing effective treatment strategies to manage acne. Acne pathophysiology is shown in Figure 1.

Key Factors Contributing to Acne Development

**Figure 2:**
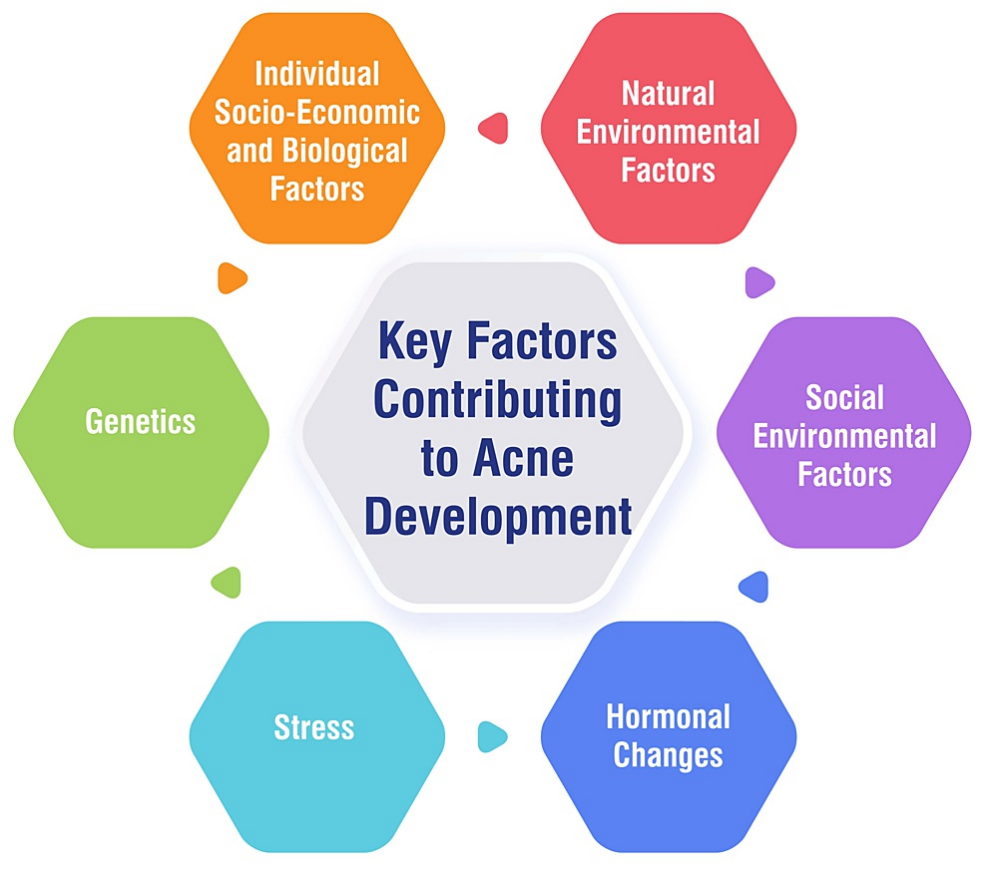
Key factors contributing to acne development. This figure is self-created by the corresponding author.

Various factors contribute to the development of acne, encompassing individual socio-economic, biological, natural environmental, social environmental, hormonal changes, stress, and genetic influences. Individual factors such as gender, age, economic status, heredity, obesity, skin type, menstrual cycle (in females), diet, smoking habits, use of cosmetics and electronic products, sleep quality, and psychological factors play significant roles in acne development [5]. Additionally, natural environmental factors like temperature and humidity can impact acne occurrence [5]. Social environmental factors, including lifestyle changes, dietary habits, exposure to air pollution, consumption of sugary foods, irregular sleep patterns, engagement in social networks, and use of social media, can also influence acne development [5]. Hormonal changes during pivotal life stages such as adolescence, pregnancy, and menopause contribute to acne formation by triggering enlarged sebaceous glands and heightened oil production in the skin [6]. Moreover, stress has been identified as a significant factor in acne development, as it can incite skin inflammation and excess oil secretion, leading to pimples and disrupting hormone levels associated with acne [6]. Finally, genetic predispositions inherited through family history can elevate the risk of acne. Individuals with a familial background of recurrent acne breakouts are more prone to genetic tendencies related to the overproduction of dead skin cells or oils [6]. These multifaceted factors collectively contribute to the complex etiology of acne vulgaris. Key factors contributing to acne development are shown in Figure 2.

Recent Advancements in Understanding Acne Pathogenesis

**Figure 3:**
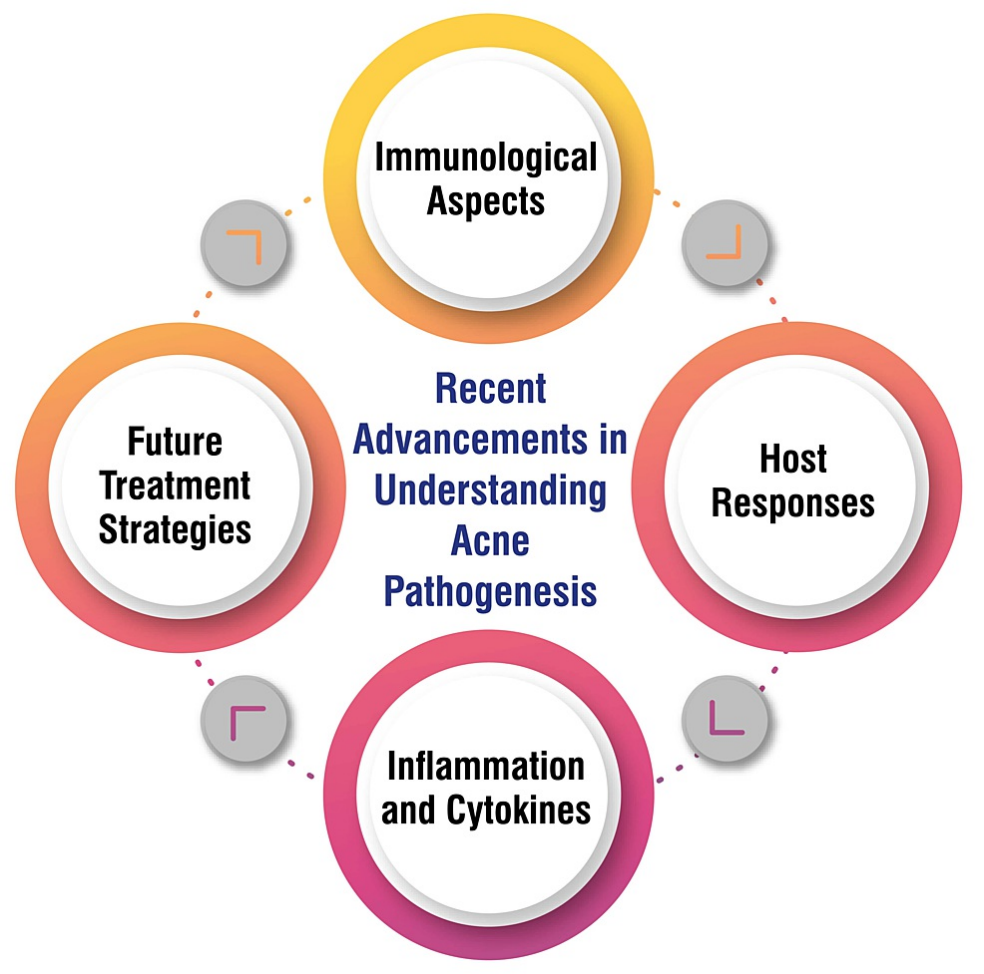
Recent advancements in understanding acne pathogenesis. This figure is self-created by the corresponding author.

Recent research underscores the significance of immunological factors in acne pathogenesis, particularly the role of *C. acnes* in stimulating inflammatory responses and cytokine production, ultimately contributing to acne lesions [7]. Host responses against *C. acnes* are crucial in acne development, with a notable shift toward a T-helper type 1 (Th1) response observed within acne lesions, indicating the intricate interplay between the immune system and acne pathophysiology [7]. Inflammation and cytokines, such as IL-1β, TNF-α, and IL-17, play pivotal roles in acne inflammation, underscoring the importance of regulating these cytokines as potential targets for acne treatments to mitigate inflammation and lesion formation [7]. Looking ahead, future treatment strategies for acne focus on innovative approaches that target immunity induction, wound healing mechanisms, and sebocyte differentiation processes to prevent acne scarring and manage acne vulgaris more effectively, offering promising avenues for therapeutic intervention [7]. These advancements highlight the evolving understanding of acne pathophysiology and the potential for novel treatment modalities to improve outcomes for individuals affected by this common dermatological condition. Recent advancements in understanding acne pathogenesis are shown in Figure 3.

Traditional treatment approaches

Topical Agents

Retinoids: Retinoids, such as adapalene, alitretinoin, tazarotene, tretinoin, and trifarotene, are commonly prescribed topical treatments available in various formulations, including creams, lotions, foams, emulsions, or gels [8]. These topical retinoids have demonstrated efficacy in treating mild to moderately severe acne by reducing the number of comedones and inflammatory lesions, making them particularly recommended as a first-line treatment for comedonal and inflammatory acne lesions [8,9]. Application of topical retinoids is advised once daily on clean, dry skin, with patients possibly needing to adjust application frequency based on skin tolerance. Initially, patients may experience skin irritation, such as peeling and redness, which generally resolves with continued use [9]. However, it's important to note that topical retinoids can lead to dryness and increased sensitivity to the sun. Patients should be counseled to apply sunscreen during the day. Additionally, due to potential teratogenic effects, topical retinoids are contraindicated during pregnancy, and caution is advised when using them in patients with eczema or young children [8]. Researchers are actively exploring novel approaches to enhance the efficacy of retinoids while minimizing side effects. This includes the development of nanotechnology-based formulations, such as solid lipid nanoparticles (SLNs) and nanostructured lipid carriers (NLCs), which offer advantages like low toxicity and high drug-loading capacity [10].

Benzoyl peroxide: Benzoyl peroxide, renowned for its potent efficacy against *P. acnes*, demonstrates superior bacteriostatic activity compared to topical antibiotics, in addition to serving as a mild comedolytic agent [11]. Offered in concentrations of 2.5%, 5%, and 10%, benzoyl peroxide is available in various formulations, including lotions, creams, gels, foams, solutions, cleansing bars, and pads [12]. Commonly reported side effects encompass skin dryness, irritation, and the potential for bleaching of clothing and bed linens, while contact allergy occurs in approximately 1 to 2 percent of users [11,12]. The application of benzoyl peroxide to clean and dry skin once or twice daily is recommended, and patients are advised about potential skin irritation and the timeframe required for visible improvement in acne lesions [11,12]. Notably, combination therapy involving benzoyl peroxide and other agents, such as erythromycin or clindamycin, has demonstrated high effectiveness in treating acne vulgaris [12].

Antibiotics: Various topical antibiotic preparations are readily available for treating skin infections, including acne vulgaris. Examples encompass bacitracin, triple antibiotic ointment (polymyxin B, neomycin, bacitracin), gentamicin, and mupirocin [13]. Mupirocin is a distinctive antibiotic derived from Pseudomonas florescens, exhibiting efficacy against Streptococcus and Staphylococcus, including methicillin-resistant *Staphylococcus aureus* (MRSA). It is offered in ointment and cream formulations and has effectively treated impetigo and secondarily infected traumatic skin lesions [13,14]. Notably, topical antibiotics like mupirocin are equally effective as orally administered erythromycin and superior to simple lesion cleaning in controlled trials. Mupirocin demonstrated equivalent efficacy to cephalexin for secondarily infected traumatic skin lesions [14]. Targeting specific bacteria responsible for skin infections like acne vulgaris, topical antibiotics aid in eradicating bacteria on the skin's surface without eliciting systemic adverse effects or toxicity [15].

Oral Medications

Oral antibiotics: Oral antibiotics, including tetracyclines like doxycycline and minocycline, as well as macrolides such as erythromycin and azithromycin, are frequently prescribed for treating acne vulgaris [14,16]. Research studies have demonstrated the efficacy of oral antibiotics, particularly doxycycline, in reducing acne lesions. Interestingly, lower subantimicrobial doses of doxycycline are equally effective as higher doses, with the added benefit of fewer adverse effects [9]. However, it is crucial to use oral antibiotics for acne for the shortest duration possible to mitigate the risk of antibiotic resistance. Typically, treatment courses may last for three to four months, although some individuals may necessitate longer durations under the supervision of a dermatologist [17]. Oral antibiotics are often employed with other medications, such as benzoyl peroxide, to enhance efficacy and diminish the likelihood of developing antibiotic resistance [18]. While severe side effects from oral antibiotics for acne are rare, increased sensitivity to sunlight is a common concern. Additionally, minocycline has been linked to skin pigmentation, mucous membranes, and teeth, whereas doxycycline can lead to photosensitivity [9,19]. Thus, carefully considering these potential adverse effects is warranted when prescribing oral antibiotics for acne management.

Oral isotretinoin: Oral isotretinoin, a systemic retinoid utilized for the treatment of severe acne, exerts its effects on sebaceous glands and keratinization processes to diminish sebum production and impede the growth of acne-causing bacteria [20,21]. Initially approved by the United States Food and Drug Administration (U.S. FDA) in 1982 for the treatment of severe, resistant, nodular acne refractory to conventional therapy, isotretinoin has also found off-label use in managing moderate acne, cutaneous T-cell lymphomas, neuroblastoma, and as a preventative measure against squamous cell carcinoma in high-risk patients [20]. Isotretinoin functions by inhibiting sebaceous gland activity and keratinization, thereby decreasing sebum production and reducing the size of sebaceous glands. Additionally, it induces apoptosis in sebocytes and mitigates the formation of comedones [21]. Orally administered as a capsule with low bioavailability, isotretinoin's absorption is enhanced with a meal. Initial dosing typically begins at 0.5 mg/kg per day and gradually escalates to 1.0 mg/kg per day over a 15- to 20-week treatment course [20]. Common side effects of isotretinoin therapy include cheilitis (dry lips), dry skin, xerostomia, heightened sun sensitivity, hypertriglyceridemia, and an elevated erythrocyte sedimentation rate. Other potential adverse effects may include pruritus, hair thinning, susceptibility to skin infections, and joint pain [20,21]. Careful monitoring and patient education regarding these potential side effects are paramount in the management of individuals undergoing isotretinoin therapy for acne.

Hormonal therapies: Hormonal therapies, such as oral contraceptive pills and spironolactone, have emerged as effective treatments for various types of acne lesions, encompassing blackheads, whiteheads, pimples, and nodules. Notably, some oral contraceptive pills have received U.S. FDA approval for their efficacy in acne treatment [22]. Spironolactone, initially prescribed for conditions like hypertension and fluid retention, has been utilized for many years to address acne and excess hair growth in women. It effectively treats deep-seated, tender acne on the lower face, jawline, or neck, especially when other treatment modalities have proven ineffective. Generally considered safe for healthy women, spironolactone can yield enhanced efficacy when used in combination with oral contraceptive pills [22]. These hormonal therapies offer viable options for individuals seeking effective management of acne, providing additional choices beyond traditional treatment approaches.

Emerging treatment modalities

Topical Therapies

Novel retinoid formulations: Novel retinoid formulations represent a promising advancement in treating acne vulgaris, with Tazarotene 0.045% lotion recently gaining approval from the U.S. FDA. This formulation demonstrates efficacy and boasts good tolerability, adding a valuable option to the array of available acne treatments [23]. Researchers have been delving into innovative delivery systems, such as liposomes, microparticles, nanoparticles, and micro-/nanofibers, to enhance retinoid solubility, stability, and targeting. These endeavors aim to overcome challenges associated with traditional retinoid formulations, including poor solubility, photosensitivity, skin irritation, and systemic side effects [24]. Moreover, advancements in nanotechnology have spurred the development of novel formulations aiming to improve the efficacy and stability of retinoids, particularly in anti-aging treatments. These nanoformulations seek to enhance penetration, reduce skin irritation, and optimize retinoid delivery for superior therapeutic outcomes [10]. Targeted topical delivery systems for retinoids offer the advantage of concentrating the active ingredient at the desired site while minimizing systemic delivery. Formulation strategies focus on enhancing thermodynamic activity, sustained release, skin barrier support, and ease of application to optimize efficacy and minimize adverse effects [25]. Notably, Conagen has pioneered the development of natural-sourced sustainable retinol derivative ingredients via precision fermentation. These unique retinoid variants offer improved stability, controlled release, and multifunctional capabilities, empowering cosmetic formulators to create products with enhanced performance and innovation in anti-aging skincare applications [26]. These advancements underscore the evolving landscape of retinoid-based therapies, offering promising avenues for improved acne treatment outcomes and anti-aging skincare solutions.

Combination therapies: Combination therapies represent a novel approach in acne management, with a triple-combination treatment consisting of clindamycin phosphate, benzoyl peroxide, and adapalene demonstrating significant efficacy in addressing moderate to severe acne. This innovative combination targets multiple pathogenic factors implicated in acne development, offering superior efficacy and tolerability compared to single or dual combinations [27]. The triple combination therapy showcases a synergistic effect, with each component contributing equally to the overall efficacy. By targeting three out of the four acne pathogenic pathways, this approach provides comprehensive coverage in acne treatment [27]. Moreover, combination therapies simplify treatment regimens for patients and promote adherence. Studies have indicated that patient adherence improves with combination agents, leading to enhanced treatment outcomes and increased patient satisfaction [27,28]. Additionally, concerns regarding antibiotic resistance are alleviated with combination therapies such as the triple combination. The incorporation of benzoyl peroxide aids in mitigating the risk of antibiotic resistance while maintaining high efficacy in managing acne vulgaris [27]. Overall, combination therapies represent a promising strategy in acne treatment, offering improved efficacy, simplified regimens, and reduced concerns regarding antibiotic resistance.

Oral Medications

New antibiotic formulations: Innovative antibiotic formulations are being explored as a potential avenue for enhancing antibiotic performance, with a novel approach based on aqueous solutions of deep eutectic solvents (DES) showing promise [29]. The quest for sustainable discovery and development of new antibiotics is underscored, focusing on leveraging innovative technologies such as artificial intelligence to expedite the identification of new antimicrobial candidates [30]. However, the need for more innovation in developing novel antibacterial treatments poses a significant concern, undermining efforts to combat antibiotic resistance. The current pipeline for new antibiotics is described as stagnant and insufficient to meet global needs [31]. Moreover, the challenge of resistance development looms large, with bacteria rapidly adapting to new drugs post-market entry. This underscores the urgent need for continuous innovation and development of effective antibiotics [31]. Bioprospecting for new antibiotics from bacterial natural products remains a labor-intensive endeavor, fraught with obstacles like the rediscovery of known compounds and low bioactivity. Efforts are therefore directed towards exploring underexplored environments for novel bacterial isolates harboring chemical novelty and bioactivity potential [32]. Encouragingly, the global pipeline of antibiotics in development exhibits promising progress, with 43 new antibiotics poised to address various resistant bacteria. Nevertheless, there remains a pressing need for additional drugs to meet current and anticipated patient needs, particularly against Gram-negative pathogens [33]. These efforts underscore the importance of innovation in antibiotic development to combat the growing threat of antibiotic resistance and safeguard public health.

Alternative systemic treatments: Alternative systemic treatments for acne encompass various remedies with purported efficacy. Vitex, a whole-fruit extract, is reputed for its effectiveness in addressing premenstrual acne by modulating hormone levels in the pituitary gland. It is believed to elevate progesterone levels while decreasing estrogen [1]. Brewer's yeast, recognized for its antimicrobial properties, is advocated by certain practitioners for acne management [1]. Furthermore, topical bittersweet nightshade, containing antimicrobial properties, is considered a viable herbal option for treating acne [1]. Manuka honey, esteemed for its antibacterial and wound-healing attributes, is promoted as an effective remedy for acne due to its properties [3]. Complementary therapies such as acupuncture, dietary modifications, and tea tree oil have been explored for their potential benefits in managing acne vulgaris [3]. However, the American Academy of Dermatology (AAD) cautions against relying solely on *all-natural supplements* for acne treatment due to inconclusive evidence of their efficacy and potential harm [1]. Limited research on alternative acne treatments has led to varying recommendations and insufficient evidence regarding their effectiveness [3]. Proponents of alternative treatments express concerns about adverse effects associated with conventional medications like antibiotics and suggest that a Western diet may contribute to acne development [3]. Despite the abundance of available alternative treatments, their safety and efficacy often need to be more adequately tested before marketing, emphasizing the importance of consulting healthcare professionals before initiating any alternative remedy [3].

Non-pharmacological Interventions

Laser and light therapies: Laser and light therapies constitute valuable treatment modalities for managing acne vulgaris, providing practical solutions for patients encountering persistent acne despite medical interventions, those experiencing adverse effects from medications, or individuals grappling with treatment adherence [34]. Nd:YAG laser treatments have effectively reduced inflammatory acne lesion counts, sebum output, and inflammatory cytokines in treated skin [34]. While this laser represents a promising modality for active acne vulgaris, further high-quality studies are warranted to confirm its efficacy [34] conclusively. Another approach involves a potassium titanyl phosphate (KTP) laser, which emits a green light pulsed beam targeting *C. acnes*, thereby inducing thermal damage to sebaceous glands [34]. Although data supporting its use in active acne are limited, studies have indicated a transient reduction in acne lesion count [34]. Light therapy, employing particles at non-thermal intensity, modulates the biological activity of the skin via different wavelengths, such as red or blue light [35]. Blue light therapy stimulates porphyrins produced by *C. acnes*, facilitating bacterial destruction and eliciting anti-inflammatory effects [35]. Conversely, red light therapy expedites wound healing, diminishes inflammation, and impedes keratinization in acne vulgaris [35]. These laser and light therapies offer promising avenues for managing acne, each tailored approach to address specific aspects of the condition, providing patients with diverse options for achieving clearer, healthier skin.

Chemical peels: Chemical peels offer a versatile approach to managing acne, with different types catering to various skin concerns and conditions. Salicylic Acid 30% Peel is frequently recommended for active acne, leveraging salicylic acid's exfoliating properties to reduce oil secretions, unclog pores, and mitigate pimple formation. Moreover, its anti-inflammatory properties effectively improve the appearance of enlarged pores [36]. Glycolic acid, an alpha-hydroxy acid commonly utilized in light chemical peels, aids in exfoliating the skin surface, reducing inflammation, and suppressing acne-related bacteria like *P. acnes* [37]. Trichloroacetic acid (TCA) treatment addresses mild acne scars by eliminating the outer layer of old skin, stimulating collagen production, and fostering smoother skin regeneration [38]. Chemical peels target acne and offer additional benefits such as refining skin texture and tone, reducing fine lines and wrinkles, and enhancing overall skin health. The selection of peel depth-whether superficial, medium, or deep-depends on factors including skin type, acne history, and desired outcomes [39]. Chemical peels are a comprehensive solution for achieving clearer, smoother, and more radiant skin by addressing various skin concerns beyond acne.

Photodynamic therapy: Photodynamic therapy (PDT) represents a minimally invasive treatment approach utilizing photosensitizers activated by light to generate reactive oxygen species, leading to targeted cell death in the treated tissue. PDT has garnered attention for its selectivity against tumor cells, rendering it effective in cancer treatment [40]. The procedure entails the administration of a photosensitizer, which accumulates in the target tissue, followed by exposure to light of a specific wavelength to activate the photosensitizer and induce cellular destruction [41]. PDT finds application in treating various conditions, including actinic keratosis, advanced cutaneous T-cell lymphoma, Barrett esophagus, basal cell skin cancer, esophageal cancer, non-small cell lung cancer, and squamous cell skin cancer [41-43]. While PDT minimizes damage to healthy cells owing to the preferential accumulation of photosensitizers in abnormal cells, it may still result in side effects such as burns, swelling, pain, and scarring in the treated area. Additionally, photosensitizers can make the skin and eyes sensitive to light following treatment [41]. Despite its potential side effects, PDT offers a valuable therapeutic option for various conditions, leveraging its targeted approach to achieve effective treatment outcomes with minimized collateral damage to healthy tissues.

Adverse effects and safety considerations

Side Effects of Traditional Treatments

Herbal medicines and complementary treatments for acne vulgaris can induce side effects such as abdominal pain, swelling, shortness of breath, nausea, pruritus, rash, skin redness, and hives [44]. It is imperative to apprise healthcare professionals of the specific type, dosage, and duration of complementary medicines being utilized to evaluate potential interactions or adverse effects [44]. Herbal medications, commonly employed for various conditions, including acne vulgaris, may elicit adverse effects such as allergic reactions, skin rashes, asthma exacerbations, headaches, dizziness, restlessness, xerostomia, seizures, fatigue, tachycardia, nausea, vomiting, and diarrhea [45]. Furthermore, certain herbal medications may interact with conventional medications, impacting their efficacy [45].

Safety Profiles of Emerging Therapies

Clascoterone, a pioneering topical androgen receptor inhibitor, has demonstrated safety and efficacy in patients aged 12 and older, exhibiting a favorable safety profile as evidenced in phase III studies [46]. This groundbreaking topical treatment represents a novel tool for dermatologists in their fight against acne vulgaris [46]. Additionally, topical retinoids are endorsed for acne treatment, supported by moderate certainty evidence from numerous studies, with safety profiles akin to vehicle treatments [46]. The external application of herbal medicines has been subject to investigation regarding its effects and safety in managing acne vulgaris, thus shedding light on alternative treatment avenues [47]. Moreover, emerging data about the safety and efficacy of spironolactone and isotretinoin challenge current conventions, suggesting a requisite for further head-to-head studies to validate their utility in acne treatment [48].

Patient Considerations and Counseling

**Figure 4:**
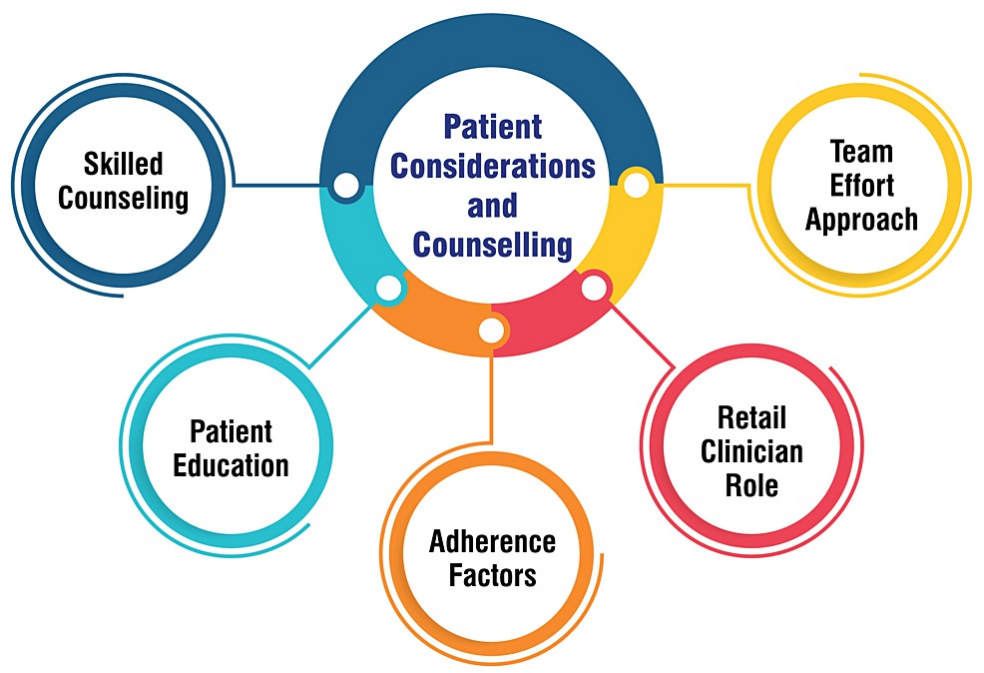
Patient considerations and counseling. This figure is self-created by the corresponding author.

Physicians must hone their counseling skills to effectively advise adolescents and adults grappling with acne vulgaris, thereby fostering improved treatment adherence [49]. Patient education plays a pivotal role in treatment success, necessitating thorough discussions about acne causes, the rationale behind prescribed medications, proper usage instructions, treatment duration, and the importance of long-term maintenance therapy [49]. Additionally, grasping patient perspectives and selecting treatment modalities aligned with their lifestyle and financial constraints are vital factors in enhancing adherence to treatment regimens [49]. Retail clinicians are significant in providing valuable guidance to individuals seeking relief from acne. They can offer counselling on steering clear of pimple popping, assisting in selecting appropriate over-the-counter products, and stressing the importance of maintaining a consistent skincare routine [50]. Embracing acne treatment as a team effort between healthcare providers and patients can prove beneficial. This approach entails managing expectations, imparting education on therapeutic options, and maintaining open communication throughout the treatment journey to forestall patient discouragement [50]. Patient considerations and counseling are shown in Figure 4.

Clinical efficacy and comparative studies

Overview of Recent Clinical Trials

A pragmatic, multicenter, phase III, double-blind, randomized controlled trial was conducted to evaluate the efficacy of oral spironolactone in adult women with facial acne. The trial revealed notable improvements in Acne-Specific Quality of Life scores at weeks 12 and 24 compared to placebo [51]. Additionally, a multicenter, double-blind, randomized, placebo-controlled study investigated the safety and efficacy of drospirenone 3 mg/ethinyl estradiol 0.02 mg in women with moderate acne vulgaris. The study aimed to assess the treatment regimen over six cycles for women with specific acne conditions [52]. Ascletis Pharma initiated a Phase III clinical trial for ASC40 (Denifanstat) targeting moderate to severe acne vulgaris in China. The trial seeks to evaluate the efficacy and safety of ASC40 compared to a placebo over 12 weeks, with treatment success and variations in lesion count serving as primary endpoints [53].

Comparative Effectiveness of Traditional vs. Emerging Treatments

The comparative effectiveness of traditional versus emerging treatments for acne vulgaris has been extensively explored in medical research. According to a network meta-analysis of 221 randomized controlled trials, oral isotretinoin emerged as the most effective treatment option, demonstrating an average reduction of 48% in the total number of lesions. Combinations of two or three medications, such as topical retinoids, topical antibiotics, oral contraceptives, and oral antibiotics, were also highly effective, resulting in reductions ranging from 25% to 36%. In contrast, single-agent therapies such as topical retinoids, oral antibiotics, and oral contraceptives were less effective, with reductions ranging from 11% to 21% [54]. Moreover, a study comparing oral isotretinoin with a 20% salicylic acid peel highlighted the efficacy of combination therapy in treating active acne [55]. Guidelines for managing acne vulgaris advocate for combination therapies and recommend standardizing the workup for patients suspected of having hormonal acne associated with polycystic ovary syndrome [56]. These guidelines underscore the importance of tailoring treatment approaches to individual patient needs and optimizing therapeutic efficacy through combination regimens, particularly in cases where conventional monotherapies may be less effective.

Future directions and challenges

Potential Advancements in Acne Treatment

Recent advancements in acne treatment have been centered on targeting inflammation, developing novel therapies, and exploring alternative approaches. Recent research underscores the significance of mitigating inflammation by addressing cytokine pathways upregulated in acne [57]. Additionally, progress in topical formulations has introduced new fixed-combination treatments such as tretinoin 0.1%/benzoyl peroxide 3% cream [58]. Moreover, cannabinoids are currently under investigation as a promising alternative therapy for acne, offering potential benefits with fewer side effects compared to traditional treatments [59]. Innovative strategies involve using energy-based devices like lasers to specifically target sebaceous glands and diminish acne lesions, ensuring safety for individuals with darker skin types [60]. Furthermore, narrow-spectrum antibiotics like sarecycline are recommended to address antibiotic resistance while minimizing disruption to the gut microbiome [60]. Long-term studies have revealed improved outcomes beyond the conventional 12-week treatment period, underscoring the necessity for sustained therapy to achieve optimal results [60]. These advancements promise to enhance acne's efficacy, safety, and overall management, signaling a positive trajectory in dermatology.

Implications for Clinical Practice and Research Priorities

Clinical practice guidelines and research studies are invaluable resources for healthcare professionals in managing acne vulgaris, offering evidence-based insights to optimize patient care. These guidelines stress the importance of evidence-based recommendations for acne treatment, advocating for benzoyl peroxide, topical retinoids, and systemic therapies like oral antibiotics or isotretinoin [61]. As the management of acne vulgaris increasingly falls within the purview of nondermatologists, understanding the diverse presentations of acne becomes crucial for tailoring individualized treatments and achieving better outcomes [62]. Research priorities in acne vulgaris management include addressing the lack of standardized methods for grading acne severity and evaluating treatment outcomes. Future research endeavors should develop consistent outcome measures to assess treatment efficacy across various clinical trials. Additionally, there is a pressing need to explore alternative therapies to counter antibiotic resistance in acne treatment and to bolster adherence to clinical practice guidelines among primary care physicians [63]. In clinical practice, healthcare providers should strive to personalize acne treatments based on the patient's clinical presentation, severity, and response to previous therapies. This personalized approach holds the potential to optimize treatment outcomes while minimizing complications. Furthermore, efforts should be directed toward raising awareness among healthcare professionals regarding the psychosocial ramifications of acne and the significance of holistic care that addresses the condition's physical and emotional facets. By adopting a comprehensive approach to acne management, healthcare providers can better meet the diverse needs of patients and improve their overall quality of life.

## Conclusions

In conclusion, this review has highlighted the recent trends in managing acne vulgaris, focusing mainly on clinical studies conducted over the last decade. It has become evident that advancements in understanding acne pathogenesis have paved the way for novel treatment modalities. By synthesizing the latest evidence, clinicians can make informed decisions regarding treatment selection, considering factors such as efficacy, safety profiles, and patient preferences. Moreover, recognizing the psychosocial impact of acne underscores the importance of comprehensive patient-centered care, including education and support. For researchers, the findings from recent studies emphasize the need for further exploration into personalized treatment approaches, combination therapies, and non-pharmacological interventions. Collaborative efforts between clinicians and researchers are essential to address the challenges of acne management, including treatment resistance and psychosocial burden. Moving forward, future research should prioritize the development of targeted therapies, long-term follow-up studies, and integration of digital health technologies to optimize patient outcomes and improve the quality of life for individuals affected by acne vulgaris.
